# Author Correction: Warning of a forthcoming collapse of the Atlantic meridional overturning circulation

**DOI:** 10.1038/s41467-025-63201-y

**Published:** 2025-08-21

**Authors:** Peter Ditlevsen, Susanne Ditlevsen

**Affiliations:** 1 Niels Bohr Institute, University of Copenhagen, Copenhagen, Denmark; 2 Institute of Mathematical Sciences, University of Copenhagen, Copenhagen, Denmark

**Keywords:** Projection and prediction, Statistics

Correction to: *Nature Communications* 10.1038/s41467-023-39810-w, published online 25 July 2023

The version of the article originally published contained two errors in the code:

1. The Strang splitting maximum likelihood estimator had a minor error (thanks to Anders Gantzhorn Kristensen for identifying it). In the last step of the Strang splitting in the pseudo maximum likelihood estimation, the flow should have been evaluated in the observation at time t_i_, but was wrongly partially evaluated at time t_i–1_. When corrected, the tipping time estimate changes by 8 years.
2. The code for calculating uniform residuals was wrong. It has no influence on estimates, only on model control.

After correcting for these errors, the estimates of tipping times and confidence intervals have changed. These changes affect Figs. 5, 6 and 7, Table 1 as well as estimates reported in the text. These have been corrected in the PDF and HTML versions of the article, as well as in the online code. The original, uncorrected version of the article is included as Supplementary information for comparison.

## Supplementary information

Original uncorrected article

