# Supplementary material for: Warning of a forthcoming collapse of the Atlantic meridional overturning circulation
Source: Nat Commun. 2023 Jul 25;14:4254. doi: 10.1038/s41467-023-39810-w (PMC10368695; doi:10.1038/s41467-023-39810-w)
Supplement: Supplementary file 1 — Supplementary Information [file 41467_2023_39810_MOESM1_ESM.pdf]

Supplementary Information for the paper

Warning of a forthcoming collapse of the Atlantic meridional overturning circulation

by Peter Ditlevsen and Susanne Ditlevsen

S1 Maximum likelihood estimators of the Ornstein-Uhlenbeck process

To obtain eq. (4) in the paper, we need the maximum likelihood estimator (MLE) of the approximate model. The approximate model is an Ornstein-Uhlenbeck (OU) process, defined as the solution to the equation

$$dX_t = -\alpha(X_t - \mu)dt + \sigma dB_t. \tag{S1}$$

This is a Gaussian process with well-known properties [1, 2]. The variance is $\gamma^2 = \sigma^2/2\alpha$ and the Δt-lag autocorrelation is $\rho = e^{-\alpha\Delta t}$. The likelihood function of the parameters given observations $(x_0, x_1, \ldots, x_n)$ is the product of the transition densities

$$L_n(\theta) = \prod_{i=1}^{n} p(\triangle, x_{i-1}, x_i; \theta) \tag{S2}$$

where $\theta = (\mu, \rho, \gamma^2)$. Here, $x_i = x(t_i)$ and $\Delta t = t_i - t_{i-1}$. The transition density is normal with conditional mean $E(X_i|X_{i-1} = x_{i-1}) = x_{i-1}\rho + \mu(1-\rho)$ and conditional variance $\gamma^2(1-\rho^2)$,

$$p(\triangle, x_{i-1}, x_i; \theta) = \frac{1}{\sqrt{2\pi\gamma^2(1-\rho^2)}} \exp\left(-\frac{(x_i - x_{i-1}\rho - \mu(1-\rho))^2}{2\gamma^2(1-\rho^2)}\right), \tag{S3}$$

see [1, 2] for details. The likelihood function is the joint probability of the observed data viewed as a function of the parameters of the statistical model, in this case discrete observations from the Ornstein-Uhlenbeck process. Considering the observed sample as fixed, the likelihood is a function of the parameters. The likelihood principle states that all the information about the parameter θ is given in the likelihood function. The maximum likelihood estimator is the value of θ which maximizes the probability of observing the given sample. In practice, the maximum of the likelihood function is found by taking the derivative with respect to the parameters (the score) and equate it to zero (the likelihood equation). For further details about likelihood theory, see any textbook in mathematical statistics, for example [3].

The maximum likelihood estimators (MLEs) derived from eqs. (S2) and (S3) are

$$\hat{\mu} = \frac{1}{n}\sum_{i=1}^{n} x_i + \frac{\hat{\rho}}{n(1-\hat{\rho})}(x_n - x_0) \approx \frac{1}{n+1}\sum_{i=0}^{n} x_i \equiv \bar{x}, \tag{S4}$$

$$\hat{\rho} = \frac{\sum_{i=1}^{n}(x_i - \hat{\mu})(x_{i-1} - \hat{\mu})}{\sum_{i=1}^{n}(x_{i-1} - \hat{\mu})^2}, \tag{S5}$$

$$\hat{\gamma}^2 = \frac{\sum_{i=1}^{n}\left(x_i - x_{i-1}\hat{\rho} - \hat{\mu}(1-\hat{\rho})\right)^2}{n\left(1-\hat{\rho}^2\right)}, \tag{S6}$$

the symbol $\hat{}$ indicates an estimator. These are obtained as follows. The score function is the vector of derivatives of the log-likelihood function with respect to the parameters. The MLE is given as solution to the likelihood equations $\partial_{\theta_k} \log L_n(\theta) = 0$, where θ_k is either μ, ρ or γ^2. The score function is

$$\begin{aligned}
\frac{\partial}{\partial\mu}\log L_n(\theta) &= \frac{(1-\rho)}{\gamma^2(1-\rho^2)}\sum_{i=1}^{n}(x_i - x_{i-1}\rho - \mu(1-\rho)), \\
\frac{\partial}{\partial\rho}\log L_n(\theta) &= \frac{n\rho}{1-\rho^2} + \frac{\sum_{i=1}^{n}(x_i - x_{i-1}\rho - \mu(1-\rho))(x_{i-1} - \mu)}{\gamma^2(1-\rho^2)} \\
&\quad - \frac{\rho\sum_{i=1}^{n}(x_i - x_{i-1}\rho - \mu(1-\rho))^2}{\gamma^2(1-\rho^2)^2}, \\
\frac{\partial}{\partial\gamma^2}\log L_n(\theta) &= -\frac{n}{2\gamma^2} + \frac{\sum_{i=1}^{n}(x_i - x_{i-1}\rho - \mu(1-\rho))^2}{2\gamma^4(1-\rho^2)},
\end{aligned}$$

whose zeros provide the MLEs in equations (S4)–(S6). It requires that $\sum_{i=1}^n (x_i - \hat{\mu})(x_{i-1} - \hat{\mu}) > 0$, otherwise the MLE does not exist.

The Fisher Information $\mathcal{I}$ of the MLEs equals minus the expectation of the Hessian $\mathcal{H}$ of the log-likelihood function. For the OU log-likelihood, the elements of $\mathcal{H}$ are given by

$$\begin{aligned}
\frac{\partial^2}{\partial\mu^2}\log L_n(\theta) &= -\frac{n(1-\rho)}{\gamma^2(1+\rho)}, \\
\frac{\partial^2}{\partial\mu\rho}\log L_n(\theta) &= \sum_{i=1}^n \left(C_1(x_{i-1}-\mu) + C_2(x_i - x_{i-1}\rho - \mu(1-\rho))\right), \\
\frac{\partial^2}{\partial\mu\gamma^2}\log L_n(\theta) &= C_3\sum_{i=1}^n (x_i - x_{i-1}\rho - \mu(1-\rho)), \\
\frac{\partial^2}{\partial\rho^2}\log L_n(\theta) &= \frac{n(1+\rho^2)}{(1-\rho^2)^2} + C_4\sum_{i=1}^n (x_i - x_{i-1}\rho - \mu(1-\rho))(x_{i-1}-\mu) - \frac{1}{\gamma^2(1-\rho^2)}\sum_{i=1}^n (x_{i-1}-\mu)^2 \\
&\quad -\frac{1+3\rho^2}{\gamma^2(1-\rho^2)^3}\sum_{i=1}^n (x_i - x_{i-1}\rho - \mu(1-\rho))^2, \\
\frac{\partial^2}{\partial\rho\gamma^2}\log L_n(\theta) &= C_5\sum_{i=1}^n (x_i - x_{i-1}\rho - \mu(1-\rho))(x_{i-1}-\mu) + \frac{\rho}{\gamma^4(1-\rho^2)^2}\sum_{i=1}^n (x_i - x_{i-1}\rho - \mu(1-\rho))^2, \\
\frac{\partial^2}{\partial(\gamma^2)^2}\log L_n(\theta) &= \frac{n}{2\gamma^4} - \frac{\sum_{i=1}^n (x_i - x_{i-1}\rho - \mu(1-\rho))^2}{\gamma^6(1-\rho^2)},
\end{aligned}$$

where $C_i, i = 1,\ldots,5$, are deterministic constants that will disappear when taking expectations. Using that $E(X_i-\mu)^2 = \gamma^2$, $E(X_i - X_{i-1}\rho - \mu(1-\rho))^2 = \gamma^2(1-\rho^2)$ and $E(X_i - X_{i-1}\rho - \mu(1-\rho))(Y_{i-1}-\mu) = 0$, we obtain the Fisher Information

$$\mathcal{I} = -E\mathcal{H} = n\begin{bmatrix} \frac{(1-\rho)}{\gamma^2(1+\rho)} & 0 & 0 \\ 0 & \frac{1+\rho^4}{(1-\rho^2)^2} & \frac{\rho}{\gamma^2(1-\rho^2)} \\ 0 & \frac{\rho}{\gamma^2(1-\rho^2)} & \frac{1}{2\gamma^4} \end{bmatrix}.$$

The inverse of the Fisher Information provides the asymptotic covariance matrix,

$$\frac{1}{n}\begin{bmatrix} \frac{\gamma^2(1+\rho)}{(1-\rho)} & 0 & 0 \\ 0 & 1-\rho^2 & 2\rho\gamma^2 \\ 0 & 2\rho\gamma^2 & \frac{2\gamma^4(1+\rho^4)}{1-\rho^2} \end{bmatrix}.$$

The diagonal elements provide the asymptotic variances of $\hat{\mu}, \hat{\rho}$ and $\hat{\gamma}^2$, respectively. For $\alpha\Delta t \ll 1$ we approximate $(1+\rho^4)/(1-\rho^2) \approx 1/(\alpha\Delta t)$ and $1-\rho^2 \approx 2\alpha\Delta t$ and obtain

$$\mathrm{Var}(\hat{\gamma}^2) \approx \frac{2(\gamma^2)^2}{\alpha T_w} = \frac{\sigma^4}{2\alpha^3 T_w}; \quad \mathrm{Var}(\hat{\rho}) \approx \frac{2\alpha\Delta t^2}{T_w},$$

where $T_w = n\Delta t$ is the observation window.

S2 Estimator of the tipping time

The process is given as solution to

$$\begin{aligned}
dX_t &= -(A(X_t-m)^2 + \lambda_t)dt + \sigma dB_t, && \text{(S7)} \\
\lambda_t &= \lambda_0(1 - \Theta[t-t_0](t-t_0)/\tau_r). && \text{(S8)}
\end{aligned}$$

and we wish to estimate the parameters $\theta = (A, m, \lambda_0, \tau_r, \sigma)$ from observations $(x_0, x_1, \ldots, x_n)$ before time t_0 and observations $(y_0, y_1, \ldots, y_n)$ after time t_0, of process X_t defined by (S7). This equation cannot

be explicitly solved, and the exact distribution is not explicitly known. A standard way to solve this is approximating the transition density by a Gaussian distribution obtained by the Euler-Maruyama scheme. However, the estimators obtained from the Euler-Maruyama pseudo-likelihood are known to be biased, especially in non-linear models [4]. Instead we use a two-step procedure: First we estimate $\alpha_0 = 2\sqrt{A|\lambda_0|}$, $\mu_0 = m + \sqrt{|\lambda_0|/A}$ and σ^2 from the stationary part before time t_0, using estimators (S4) – (S6), where $\alpha_0 = -\log(\rho)/\Delta t$ and $\sigma^2 = 2\alpha_0\gamma^2$. This yields estimates $\lambda_0(A) = -\alpha_0^2/4A$ and $m(A) = \mu_0 - \alpha_0/2A$ as a function of parameter A and the estimated parameters. The two remaining parameters A and τ_r are then estimated from the data after time t_0, where we no longer can use the OU process, since the linear approximation breaks down when the tipping point is approached. Simplifying by assuming that λ is constant between observations, i.e., piecewise constant and jumping every month where new AMOC observations are available, we obtain transition densities that are non-linear transformations of Gaussian densities, making the inference problem tractable as follows. We use a pseudo-likelihood induced by the Strang splitting scheme, shown to be robust for highly non-linear models [4]. Consider the two subsystems

$$dX_t^{(1)} = -\alpha(\lambda)(X_t^{(1)} - \mu(\lambda))dt + \sigma dB_t, \tag{S9}$$

$$dX_t^{(2)} = -A(X_t^{(2)} - \mu(\lambda))^2 dt, \tag{S10}$$

where $\alpha(\lambda) = 2\sqrt{A|\lambda|}$ and $\mu(\lambda) = m + \sqrt{|\lambda|/A}$. The drift of subsystem (S9) is the Taylor expansion of the drift in eq. (S7) to first order around the fixed point $\mu(\lambda)$ and is an OU process, of which we know the distribution and the likelihood (see S1). Eq. (S10) is a deterministic equation with the non-linear part, which solution is also known. We obtain the following two flows:

$$\phi_\Delta^{(1)}(x) := (X_{t+\Delta}^{(1)} \mid X_t^{(1)} = x) = xe^{-\alpha(\lambda)\Delta} + \mu(\lambda)(1 - e^{-\alpha(\lambda)}) + \xi_t$$

$$\phi_\Delta^{(2)}(x) := (X_{t+\Delta}^{(2)} \mid X_t^{(2)} = x) = \frac{\mu(\lambda)A\Delta(x - \mu(\lambda)) + x}{A\Delta(x - \mu(\lambda)) + 1}$$

where $\xi_t \sim N(0, \Omega_\Delta)$, $\Omega_\Delta = \frac{\sigma^2}{2\alpha(\lambda)}(1 - e^{-2\alpha(\lambda)\Delta})$.

The Strang splitting [4] then approximates by

$$(X_{t+\Delta} \mid X_t = x) = \left(\phi_{\Delta/2}^{(2)} \circ \phi_\Delta^{(1)} \circ \phi_{\Delta/2}^{(2)}\right)(x) = \phi_{\Delta/2}^{(2)}\left(e^{-\alpha(\lambda_t)\Delta}\phi_{\Delta/2}^{(2)}(x) + \mu(\lambda_t)(1 - e^{-\alpha(\lambda_t)\Delta}) + \xi_t\right), \tag{S11}$$

which is defined for all $x > \mu(\lambda_t) - 2/A\Delta$. Since we are only interested in simulating the process up to the time where X_t crosses the separatrix between the two attractors, which happens for $x < m$, we require that $m > m + \sqrt{|\lambda_t|/A} - 2/A\Delta \geq m + \sqrt{|\lambda_0|/A} - 2/A\Delta$, i.e., $\Delta < 2/\sqrt{A|\lambda_0|} = 4/\alpha_0$. This is always fulfilled, since $\Delta = 1/12$ and α_0 is estimated to be less than 4.

The transition density (S11) is a nonlinear transformation of a Gaussian random variable, leading to the pseudo-loglikelihood function (up to a constant)

$$-\log L_n(A, \tau_r) = \frac{1}{2}\sum_{i=1}^n \log(\Omega_\Delta) + \sum_{i=1}^n \frac{Z_i^2}{2\Omega_\Delta} - \sum_{i=1}^n \log\left|\frac{d}{dx}(\phi_{\Delta/2}^{(2)})^{-1}(y_i)\right| \tag{S12}$$

where

$$Z_i = (\phi_{\Delta/2}^{(2)})^{-1}(y_i) - e^{-\alpha(\lambda_{t_{i-1}})\Delta}\phi_{\Delta/2}^{(2)}(y_{i-1}) + \mu(\lambda_{t_{i-1}})(1 - e^{-\alpha(\lambda_{t_{i-1}})\Delta}),$$

see [4] for details. The first two terms in (S12) are the standard terms from a Gaussian likelihood, the last term originates from the non-linear transformation. Estimates of parameters A, τ_r are then obtained by minimizing (S12). Since division by A enters the calculations of λ_0 and m and thus the pseudo-likelihood, estimates are sensitive to small values of A. We therefore regularize the optimization problem by adding a penalization term on small values of A. The term $-pn(1/A - 1)$ is added to (S12) for $A < 1$, where $p \geq 0$ is a penalization parameter determined by cross-validation on simulated data sets by minimizing the mean squared distance between the estimated ramping time on each data set to the value of the ramping time used in the simulation. The optimal value was $p = 0.004$.

The parameter estimates are found numerically by minimizing $-\log L_x(\theta)$. For this we apply the optimizer `optim` in R, using the Nelder-Mead algorithm.

Confidence intervals are obtained by parametric bootstrap: 1000 repetitions of the model are simulated with the estimated parameters, and on each synthetic data set, parameters are estimated. The empirical quantiles of the 1000 estimates thus obtained are used to construct confidence intervals.

References

[1] Ditlevsen, S., Cencerrado Rubio, A. & Lansky, P. Transient dynamics of Pearson diffusions facilitates estimation of rate parameters. *Communications in Nonlinear Science and Numerical Simulation* **82**, 105034 (2020).

[2] Forman, J. L. & Sørensen, M. The Pearson diffusions: A class of statistically tractable diffusion processes. *Scandinavian Journal of Statistics* **35**, 438–465 (2008).

[3] Lauritzen, S. *Fundamentals of Mathematical Statistics* (Chapmann & Hall, 2023).

[4] Pilipovic, P., Samson, A. & Ditlevsen, S. Parameter estimation in nonlinear multivariate stochastic differential equations based on splitting schemes. *arXiv:2211.11884* (2022).
